# Pneumonia due to a Rare Pathogen:* Achromobacter xylosoxidans*, Subspecies* denitrificans*

**DOI:** 10.1155/2017/3969682

**Published:** 2017-08-15

**Authors:** Hesham Awadh, Munthir Mansour, Obadah Aqtash, Yousef Shweihat

**Affiliations:** ^1^Internal Medicine Department, Marshall University Joan C. Edwards School of Medicine, Huntington, WV, USA; ^2^Pulmonology Department, Marshall University Joan C. Edwards School of Medicine, Huntington WV, USA

## Abstract

*Achromobacter xylosoxidans*, subspecies* denitrificans*, is a gram-negative rod recently implicated as an emerging cause of infection in both immunosuppressed and immunocompetent populations. Few cases are reported in literature involving multiple body systems. Diagnosis depends on cultures of appropriate specimens, and management usually is by administration of appropriate antibiotics (usually agents with antipseudomonal activity). We report a rare case of pneumonia due to infection with this organism, in a patient with preexisting bronchiectasis secondary to chronic aspiration.

## 1. Introduction

*Achromobacter xylosoxidans* subspecies* denitrificans* is a gram-negative bacillus recently emerging as a causative agent of infection [1]. The* Achromobacter *species has many subspecies the most clinically important of which are* xylosoxidans* and* denitrificans* [1]. There are infrequent reports of infections with this organism involving various organs (Table 1). It seems that a dysfunctional immune status and/or prior structural damage plays a role in the pathogenicity* Achromobacter xylosoxidans, *subspecies* denitrificans*. We report a rare case of pneumonia due to this organism in a 45-year-old female with bronchiectasis secondary to recurrent aspiration.

## 2. Case Presentation 

**Figure 1:**
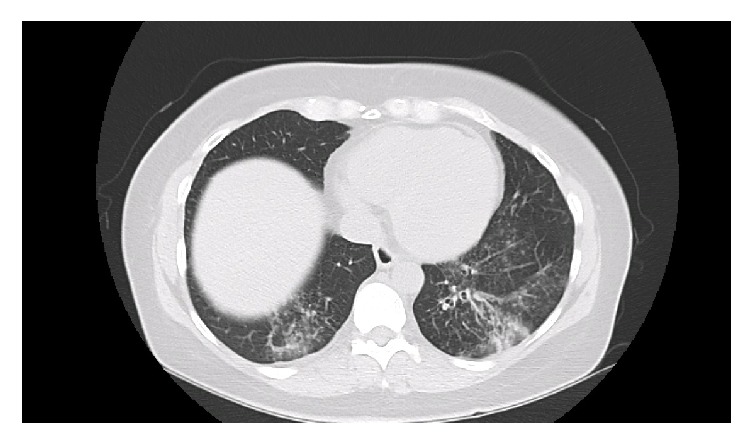
Infiltrates and bronchiectatic changes in lower lobes bilaterally.

This is a 45-year-old White female with past medical history of asthma and gastroesophageal reflux disease (GERD) treated with Nissen fundoplication in the past. She presented to our clinic with chronic cough productive of greenish sputum. She improved with previous antibiotic use of levofloxacin on several occasions but her symptoms would recur as soon as she stops the antibiotics. Chest X-ray at initial evaluation showed an infiltrate bilaterally more pronounced on the right lower lobe. A CT scan of the chest was obtained and confirmed the infiltrates and showed bronchiectatic changes bilaterally in the lower lobes (Figure 1). Her autoimmune screen came back negative for rheumatoid arthritis and Sjogren's syndromes, yet her immunoglobulins were elevated and her alpha one antitrypsin and immunoglobulin E (IgE) were at normal levels. Her sweat chloride test was normal. Chronic recurrent aspiration was suspected and an esophageal PH monitor along with esophageal manometry confirmed our suspicion of chronic aspiration secondary to severe acid reflux with elevated DeMeester score. Bronchoscopy was performed to rule out an obstructive disease and to obtain samples to rule out mycobacterial disease. Cultures came back positive for heavy growth of a nonfermenter later identified as* Achromobacter xylosoxidans, *subspecies* denitrificans*. Sensitivities were evaluated and the bacteria was sensitive to levofloxacin, amikacin, cefepime, ceftazidime, gentamicin, meropenem, piperacillin/tazobactam, tobramycin, and trimethoprim-sulfamethoxazole. It was found to be resistant to aztreonam, cefotaxime, and ciprofloxacin, with incubation period of 5 days. Airway clearance techniques with percussion and flutter valve and bronchodilator therapy with hypertonic saline nebulizers were initiated. She was started on a 3-week course of levofloxacin but her symptoms recurred one month after stopping the antibiotic despite airway clearance techniques. She was started again on levofloxacin for two more weeks with good clinical response and no recurrence of symptoms after a total 5 weeks of antibiotic therapy. She was referred for surgical intervention to abolish the ongoing injury to the airway and stop the aspiration insult to the airways.

## 3. Discussion

*Achromobacter denitrificans* is an aerobic, nonglucose fermenter gram-negative bacillus and flagellated and motile and produces acid from xylose [1]. The genus* Achromobacter *has multiple subspecies:* xylosoxidans, ruhlandii, piechaudii*,* denitrificans, spanius, insolitus,* and* marplatensis*. The most clinically significant subspecies are* Achromobacter xylosoxidans* and* denitrificans *[2]. It can be found in nature in soil, and the* xylosoxidans* subspecies has an affinity for aquatic surfaces. There are more clinically significant isolates of the subspecies* xylosoxidans* compared to* denitrificans* in terms of incidence of infection and clinical variety. Reports about infections with* Achromobacter denitrificans* are rare as an emerging pathogen.

There is a multitude of respiratory system infection cases due to* Achromobacter xylosoxidans*, subspecies* xylosoxidans* but not* denitrificans* [3]. The first reported pneumonia case due to* Achromobacter denitrificans* was reported from India in a 48-year-old male clerk in a chemical factory. It was isolated from sputum at two different occasions with no other concomitant isolates. It was sensitive to meropenem, imipenem, piperacillin-tazobactam, ticarcillin, trimethoprim-sulfamethoxazole, and third-generation cephalosporins. It was successfully treated with two weeks of meropenem [4]. Our case is to date the second reported case of* Achromobacter xylosoxidans* subspecies* denitrificans*. Other reported infections in adults include meningitis [5], endocarditis [6], endocarditis with aortic root abscess [7], renal abscess [8], peritoneal dialysis catheter related peritonitis [9], and exit site infection [10] (Table 1).

Diagnosis depends on isolation of the organism depending on site of the infection. So far, we have reported isolates from septum, pus, peritoneal fluid, and cerebrospinal fluid (Table 1). Identification can be via standard culturing methods. In our case bronchoalveolar lavage samples have been incubated in Blood Agar (TSA with 5% Sheep Blood)/MacConkey Agar Plate using a BioMérieux VITEK-2 system, incubated at 36-37 degrees Celsius.

The immunosuppressed population are at higher risk of infection due to* Achromobacter* species [3], yet as seen in Table 1 most of the patients had not been overtly immunocompromised but may have had predisposing conditions (end stage renal disease, presence of catheters, etc.). Both cases with involvement of respiratory system had a background of bronchiectasis. The former [4] was most likely secondary to tuberculosis while in our case it is most likely secondary to recurrent aspiration. We theorize that the damaged bronchiectatic lung tissue predisposed to the infection with this organism. This had been illustrated before in that* Achromobacter *species is known to colonize and infect cystic fibrosis patients [11]. In the other reported cases (Table 1), we can notice that structural damage and/or foreign body had been present: previous trauma, prosthetic valve, peritoneal dialysis catheter, and renal stones. Pathogenicity of* Achromobacter* species has been previously studied [12] with demonstrated ability to form biofilms and motility (via pili and flagella) which can potentiate infections in setting of structural damage and devices.

Management depends on administration of appropriate antibacterial agents, yet duration of treatment is not exactly defined due to lack in specific guidelines in this regard. The cases reported so far (Table 1) had been managed with different regimens but the most palpable response was to carbapenems with durations ranging from 2 to 14 weeks (Table 1). The sensitivity of the isolates is outlined below in Table 1, but the* Achromobacte*r species had been historically responsive to antipseudomonal agents [1] with various success rates depending on site of infection and complexity of the cases. Our patient was cured after 5 weeks of oral levofloxacin therapy (after initial failure after three weeks). The outcome is generally excellent with clearance of the infection.

## 4. Conclusion

Rare causes of pneumonia should be investigated since appropriate detection can facilitate accurate antibacterial management. We theorize that structural damage (bronchiectasis secondary to chronic aspiration) plays a role in the pathogenesis of pneumonia in our patient. Combined management with antibiotics and airway clearance techniques resulted in an excellent outcome.

## Figures and Tables

**Table 1:** Previous reports of infections with *Achromobacter xylosoxidans subspecies denitrificans*, detailing type of infection, antimicrobial sensitivity, and duration of treatment.

Year reported	Type of infection	Isolation specimen	Comorbid conditions	Sensitivity	Antibiotic of choice	Treatment duration
2011 [6]	Prosthetic valve endocarditis	Blood	Tetralogy of Fallot	Cefepime, ceftazidime, ciprofloxacin, imipenem, levofloxacin Piperacillin/tazobactam Ticarcillin/clavulanic acid, trimethoprim-sulfamethoxazole	Piperacillin/tazobactam (not specified), then imipenem	6 weeks of piperacillin/tazobactam, then 8 weeks of imipenem

2011 [5]	Meningitis	Cerebrospinal fluid	Prostatic adenocarcinoma Epilepsy Hyperlipidemia Atrial fibrillation Remote history of cranial trauma	Not reported	Meropenem 2 g intravenously (IV)	15 days

2014 [9]	Peritoneal dialysis catheter Exit site infection	Pus collected from exit site	Diabetes mellitus, chronic kidney disease (near end stage renal disease)	Not reported	Ciprofloxacin 250 mg every twelve hours	14 days

2012 [8]	Right renal abscess with renocutaneous fistula	Pus collected from intrarenal abscess	Hypertension, chronic kidney disease, benign prostatic hyperplasia Recurrent bilateral nephrolithiasis	Colistin, imipenem, meropenem, piperacillin/tazobactam	Meropenem 1 g IV	60 days

2013 [7]	Prosthetic valve endocarditis with aortic root abscess	Blood	Congenital aortic stenosis, history of aortic valve valvotomy	Not reported	Meropenem, trimethoprim- sulfamethoxazole, then levofloxacin	4 weeks

2014 [4]	Pneumonia	Sputum	History of tuberculosis	Meropenem, imipenem, piperacillin/tazobactam, ticarcillin, trimethoprim-sulfamethoxazole, third-generation Cephalosporins	Meropenem 1 g q 8 hours	2 weeks

2014 [9]	Peritoneal dialysis Catheter related peritonitis	Effluent dialysate	End stage renal disease on peritoneal dialysis	Ciprofloxacin (other antibiotics not specified)	Ciprofloxacin (not specified)	Duration not specified

2017	Current case Pneumonia / Bronchiectasis	Bronchoalveolar lavage	Gastroesophageal reflux disease, asthma	Amikacin, cefepime, ceftazidime, gentamicin, levofloxacin, meropenem, piperacillin/tazobactam, tobramycin, trimethoprim/sulfamethoxazole	Levofloxacin	6 weeks
